# A Novel Episcleral Macular Buckling: Wire-Strengthened Sponge Exoplant for Recurrent Macular Hole and Retinal Detachment in High Myopic Eyes

**Published:** 2013

**Authors:** Hassan A. Mortada

**Affiliations:** Department of Ophthalmology, Kasr El Aini Hospital, Cairo University, Cairo, Egypt

**Keywords:** Episcleral Macular Buckling, Macular Exoplant, Macular Hole, Recurrent Retinal Detachment, Macular Hole Closure, High Myopia

## Abstract

The purpose would be to describe and evaluate a novel technique of episcleral macular buckling in postvitrectomy recurrent macular hole retinal detachment in highly myopic eyes. A 7mm silicone sponge strengthened with a U-shaped 0.5mm orthodontics stainless steel wire fed along its length and hand-bent to produce L-shaped buckle of appropriate shape and length, is used. The episcleral macular buckling has performed on 15 highly myopic eyes (axial length > 30mm) with recurrent macular hole retinal detachment following silicone oil removal. Buckle localization has been performed by manipulating the long arm of the exoplant, under direct internal visualization, scleral marking and suturing. Successful retinal reattachment with improvement in visual acuity achieved in all 15 eyes. Closure of the macular holes was confirmed by Optical Coherence Tomography. The anatomical and functional outcomes of this new technique of macular buckling appears to provide an effective and feasible means of retinal reattachment and hole closure in postvitrectomy recurrent macular hole detachment in highly myopic eyes.

## INTRODUCTION

Episcleral Macular Buckling (EMB) [1-6] has been considered as a standard surgical treatment for the repair of retinal detachment caused by macular hole in highly myopic eyes prior the introduction of Pars Plana Vitrectomy (PPV) [7] and several additional procedures later on [8-22]. The current regained interest in EMB is supported by recent solid evidence of comparable or even better outcomes with EMB over PPV [23-26]. In 1980, Ando introduced the Ando plomb, [27,28] that has shown long-term success as a primary EMB, and recently for postvitrectomy recurrent cases with a modified technique [29]. However, the Ando plomb is not commercially available in all countries, and it might not be practical to order the implant for each patient, as it comes in different sizes, while this procedure is not regularly performed as a continuous practice. Theodassiadis [24], and Ripandelli [25] both reported EMB success with sponge and solid silicone exoplant respectively, however the described surgical techniques could be still considered challenging. In this study we are describing a new exoplant that could be easily assembled, using readily available materials, in the operating theatre. We are reporting our experience with this exoplant in eyes with post-vitrectomy recurrent retinal detachment due to macular hole in highly myopic eyes, following silicone oil removal.

## METHODS

**Exoplant Assembly**

**Figure 1:**
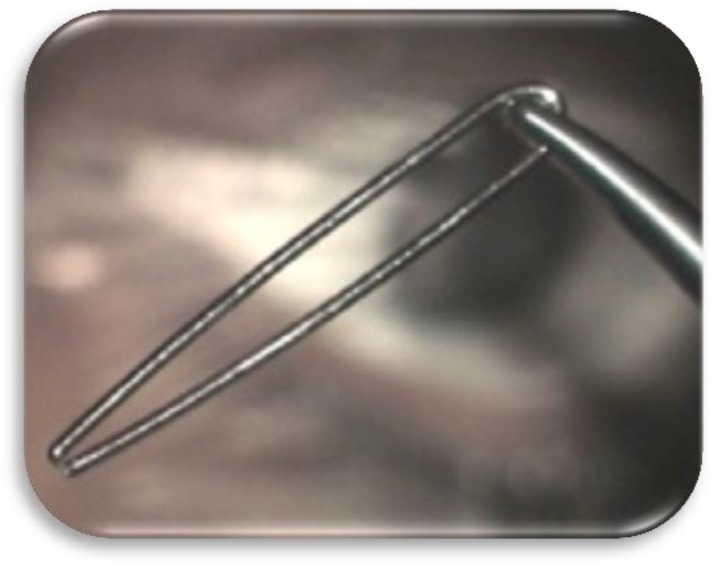
U-shape, 0.5mm orthodontic stainless steel wire bent with 131 pliers.

**Figure 2:**
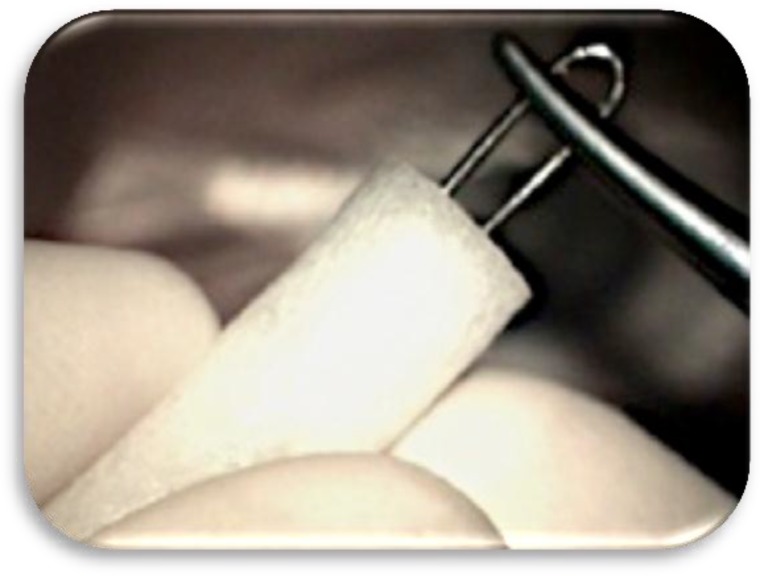
Wire is fed inside a 7mm sponge.

**Figure 3:**
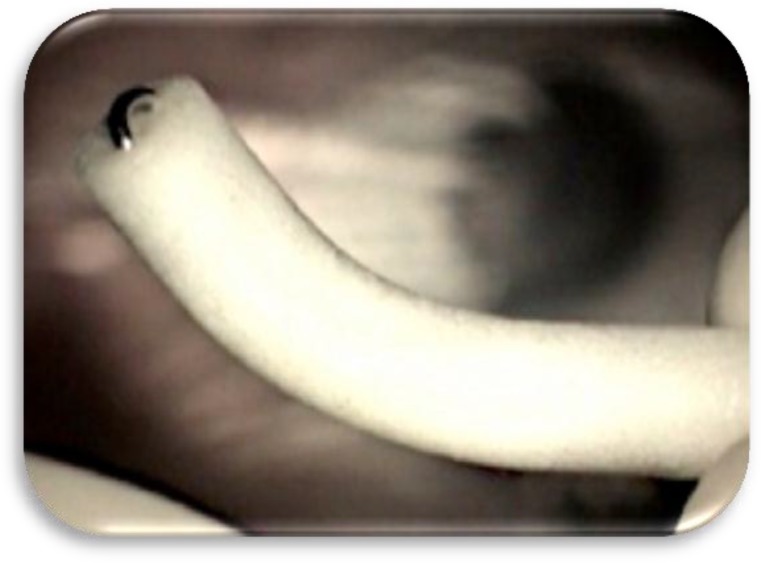
The exoplant bent by hand for the proper configuration to have a short arm for macular indentation and a long arm for manipulation.

A 7mm silicone sponge exoplant cut for the proper length for each eye (35 – 45mm) is used. A 0.5mm orthodontic steel wire that is originally used for dental braces is used to strengthen the silicone sponge. The wire firstly bent, using 131 pliers, into a U-shaped at the middle, with 2mm separation between the 2 arms (Figure 1). This will strengthen the wire while being fed inside the sponge and the 2mm width will give little room for rotation of the sponge over the sclera during suturing. The U-shaped wire has inserted in the middle of the sponge, and fed through its whole length (figure 2). As necessary, the exoplant bent by hand for the proper configuration to fit with each globe, having a shorter arm for the macular buckle and a longer arm for its manipulation and later suturing (figure 3). 

**Figure 4:**
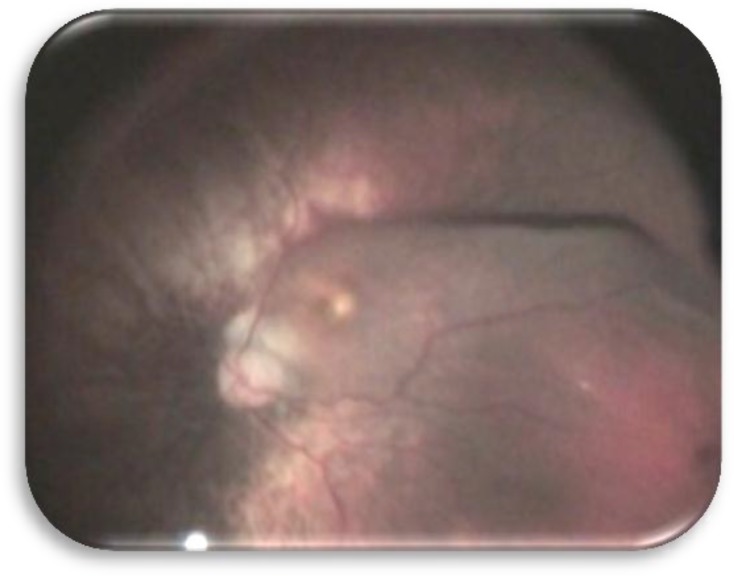
Internal visualization of the indentation effect of the exoplant intraoperatively for proper localization and suturing

**Figure 5a:**
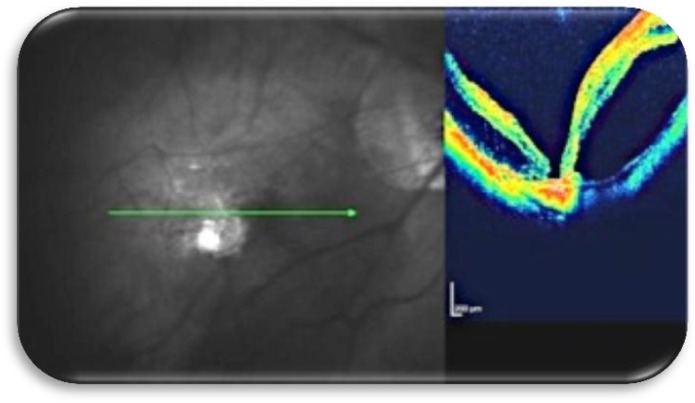
Fundus photograph and macular OCT with recurrent retinal detachment and myopic macular hole before surgery

**Figure 5b:**
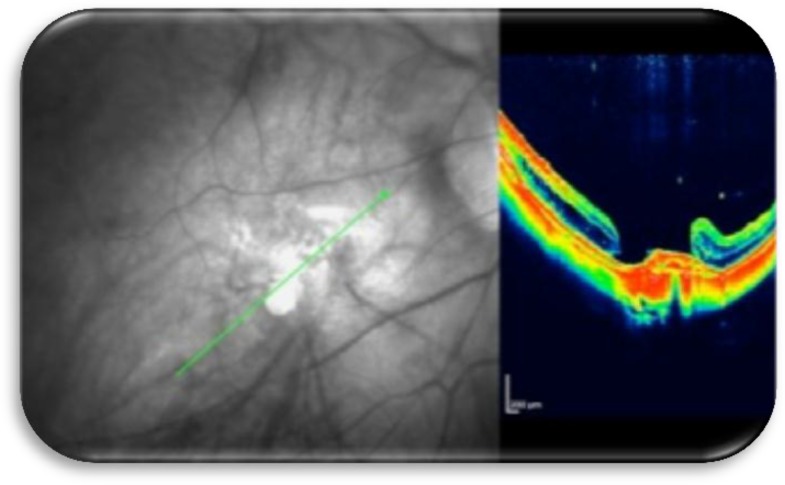
Fundus photograph showing the buckle effect supporting the macular area and OCT showing closure of the macular hole.

**Figure 6:**
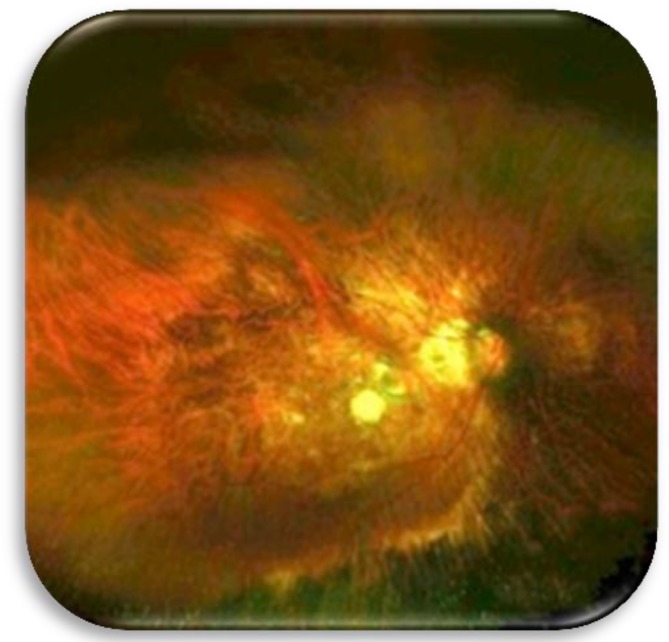
Postoperative wide angle picture showing the buckling at the posterior pole

**Surgical technique**

The lateral, superior and inferior recti were exposed through a lateral periotomy and traction sutures were applied. Disinsertion of the lateral rectus was not necessary in any case. The episcleral tissue was dissected and the surface of the sclera was exposed in a broad area extending towards the posterior pole of the eye to facilitate insertion and suturing of the exoplant. Using a 23-gauge vitrectomy system, the cannula system was inserted and the infusion line connected. A chandelier light was used. A revision of the vitrectomy was performed using Triamcinolone acetinoide to look for any residual adherent posterior cortical vitreous. The Tano scraper was helpful in this respect. A small peripheral retinotomy was performed, just posterior to the ora, usually in upper temporal quadrant. Perfluorocarbon liquid was then slowly injected to displace the subretinal fluid to be drained through the peripheral retinotomy until complete retinal reattachment could be achieved. The cannulas then plugged and the intraocular pressure adjusted at 10mmHg. Macular buckling is then performed under direct internal visualization using the chandelier light. With the assistant exerting traction on the superior and inferior recti to steady the globe, the long arm of the exoplant grasped with a broad utility forceps, and introduced along the inferior border of the lateral rectus, pushing the short arm towards the posterior pole. The manipulation of the exoplant directly monitored until the short arm has been seen under the macular area (figure 4). The assistant marked the position of the long arm on the sclera. Two parallel lines marked on the sclera identifying the sides of the exoplant and a third perpendicular line on the sclera and exoplant to mark the antero-posterior position. Two mattress scleral sutures were taken, with slipknot, to fix the long arm to the marked site. The position of the buckle revised and if proved to under the posterior pole, the sutures were tied permanently. Endolaser was applied to the peripheral retinotomy. Finally, PFCL was exchanged with silicone oil or SF6.

## DISCUSSION

Although the exact pathogenesis remains unclear, macular holes are more frequently accompanied by retinal detachment in highly myopic eyes than in those with lesser or no myopia and the incidence of retinal detachment increases with the presence and the degree of posterior staphyloma [30-32]. Several forces are acting on the posterior pole in a pathological myopia: Adherent or partially detached posterior hyaloid traction with or without vitreoschisis, altered internal limiting membrane ,potent (rigid) retinal arteriole, and rogression of the posterior staphyloma. The first 3 factors exert traction to detach or split the retina and can be relieved by PPV, posterior hyaloid and ILM peeling. The fourth factor pulls the posterior global wall away from the overlying retina. This last factor can only be corrected by macular buckling. Which of these factors plays the major role in production of myopic macular retinoschisis and macular hole retinal detachment, has not definitely understood. The effect of each of these factors may vary from case to case. In cases of myopic macular hole retinal detachment with posterior staphyloma, it may therefore be beneficial to perform macular buckling as a means of bringing the posterior global wall back into proximity with the retina, thus promoting retinal reattachment. This may help to correct the four forces acting on the posterior pole in high pathological myopia.

Various EBM explants have been proposed for this purpose and have, in some cases, been found to be effective [1-6]. However, a number of surgeons have noted that the procedure is difficult to perform and particularly that direct suturing of the exoplant to the posterior pole of the eye is very difficult and may prove to be hazardous.

Since its introduction in 1982, PPV for myopic macular hole retinal detachment [7] has gained popularity with several additional procedures such as intraocular gas tamponade [8-14], silicone oil tamponade [12,15], laser photocoagulation [12,16], transscleral diathermy[17], and internal limiting membrane peeling [18-22]. For some time, it was considered the preferred treatment of this relatively complicated retinal detachment, and EMP seemed to be outdated mainly due to its challenging technique. However, today the treatment of choice between PPV and EMB is once again debatable, due to the recent solid evidence of comparable or even better long-term visual and anatomical outcomes of EMB over PPV [23-26].

Ando innovated EMB by introducing a specially shaped, rod-form macular exoplant, referred to as the Ando plomb, which was smaller, more easily fashioned and surgically less challenging than earlier exoplants [27,28]. Ando plomb (exoplant) length and shape facilitate suturing to the sclera, and its wire-reinforced semi-rigid structure enables adjustment of the short arm inclination for optimal positioning of the indenting head against the staphyloma area. The surgical technique was recently modified by Ando [29] by incorporating episcleral tissue removal and intraocular pressure control. Although Ando plomb was initially introduced as a primary procedure for these cases, recently it showed equal success with postvitrectomy recurrent cases. However, as mentioned earlier, this implant is not available commercially everywhere, and we have experienced difficulties in obtaining it in Egypt.

Theodassiadis and Ripandelli, [24, 25] both described EMB success with sponge and solid silicone exoplant respectively; however the techniques described are still challenging. Both techniques required disinsertion of the lateral rectus muscle and exposure of the posterior pole with direct suturing to this critical area in case of Ripandelli technique.

In this report, we described an exoplant for macular buckling that could be easily assembled in the operating theatre, and could perform very similarly to Ando plomb. The stainless steel wires used in orthodontic procedures forming the wire of dental braces. The fed wire inside the silicone sponge, as described previously, strengthens it and maintains its shape. The wires permit manual bending to obtain proper configuration and optimal positioning of the sponge on the posterior pole and ensure long-term retention of this configuration after implantation. It also permits internal visualization of the buckle for proper localization before suturing.

Our experience with this exoplant in 15 eyes suggests that this technique should be considered for inclusion wherever possible in the implementation of macular buckling. The procedure employed apparently promoted macular hole closure as seen clinically and on OCT images (figure 5a, 5b, 6), even though the eyes had previously underwent vitrectomy, Triamcinolone acitenoide assisted peeling of adherent posterior cortical vitreous, internal limiting membrane peeling and silicone oil tamponade for 6 months. As our initial experience with this type of EMB, silicone oil used as tamponade in the first 5 eyes. SF6 used as tamponade in the following 10 eyes. In all eyes the hole remained closed and the retina attached following silicone oil removal (after 3 months) and absorption of SF6. This suggests that macular buckling procedure performed with the above mentioned technique promoted macular hole closure and retinal reattachment in highly myopic eyes, with a resultant improvement in visual acuity.

Advantages of this technique could be defined as:

1. The use of readily available material (dental braces stainless steel wire and the silicone sponge) to assemble the exoplant , making it more feasible and practical. 2. Direct visualization of the buckle during positioning underneath the macular hole with proper localization thus avoiding the preplaced sutures and re-suturing. The longer arm of the exoplant allows its manipulation at a distance. 3. No need for exposing the posterior pole for direct buckle suturing which may prove to be difficult and hazardous. The bent-head (short arm) can provide a good macular support with the sutures placed on the longer arm further away.

Disadvantage could be defined as the large size of the exoplant (7 mm sponge) may lead to a mild to moderate ocular motility limitation on lateral gaze position.

As demonstrated in our work and in other reports, [23-26], episcleral macular buckling can provide good anatomic results, macular hole closure, stable retinal attachment and improved visual acuity.
